# 187. Risk Factors and Outcomes of Patients with Serratia Bacteremia at an Academic Medical Center

**DOI:** 10.1093/ofid/ofad500.260

**Published:** 2023-11-27

**Authors:** Katherine L Doktor, Madeline King, Diana Martirosyan, Scott R Moore, Henry S Fraimow

**Affiliations:** Cooper Medical School of Rowan University, Camden, New Jersey; Cooper University Hospital, Camden, New Jersey; Cooper University Hospital, Camden, New Jersey; Sparrow Hospital, Grosse Pointe Woods, Michigan; Cooper University Hospital and Cooper Medical School of Rowan University, Camden, NJ

## Abstract

**Background:**

The majority of reported *Serratia* infective endocarditis (SIE) in recent years are healthcare-associated. At our urban institution with a high prevalence of people who inject drugs (PWID), we noted an increasing number of *Serratia* bacteremias (SB) and SIE over the past decade. Our study describes patient factors associated with SB and SIE.

**Methods:**

We evaluated charts of patients with SB at our institution between 2010 and 2022 to determine risk factors and clinical outcomes.

**Results:**

There were 157 episodes of SB in 153 patients. The majority were male (57%), white (57%) with a mean age 53. The most common source of infection was catheter- line-associated infection (CLABSI) (27%), followed by primary bacteremia (19%). Twenty-six % were PWID with younger mean age (41 years). PWID had lower mortality than non-PWID (23% vs 44%, p=0.023). Sixty-nine (46%) were admitted to the intensive care unit (ICU) with significantly higher mortality associated with ICU admission (48% vs 8%, p=0.015).

Echocardiograms were performed in 59% (93/157) of SB episodes and 14% had SIE (13/93). Most SIE (69%) occurred after 2017 with 89% in PWID (8/9). The most common comorbidity with SIE was injection drug use (IDU) (69%). All non-IDU SIE were CLABSI-related; 75% had hemodialysis catheters. Tricuspid valves were infected in 46%. Sixty-two % of those with SIE had septic emboli. Most SIE (69%) required ICU admission. Overall mortality was 31%.

Only 2% (3/153) of patients with SB developed antibiotic resistance on therapy. Clinical worsening or failure occurred in 20% (32/157). Only 5% (7/157) had microbiologic recurrence within 30 days. 32% of discharged patients re-presented to the hospital within 30 days. Almost all deaths (92%) occurred during initial admission.

**Conclusion:**

Patients with SB were quite ill with a high rate of in-hospital mortality; over half of the patients had antibiotic exposures prior to developing SB or presented within 90 days of a prior hospitalization. The majority of SB were line-associated, including all episodes of non-IDU SIE. IDU was the second most common comorbidity and PWID with SB had a higher rate of SIE, with most IDU-associated SIE diagnosed after 2017. This aligns with other recent studies documenting the increased prevalence of SB, especially in PWID.

**Disclosures:**

**Madeline King, PharmD**, La Jolla: Advisor/Consultant|Shionogi: Advisor/Consultant

